# Modeling epileptic dynamics in the hippocampus using a multiscale approach

**DOI:** 10.1186/1471-2202-14-S1-P194

**Published:** 2013-07-08

**Authors:** Sébastien Naze, Christophe Bernard, Viktor Jirsa

**Affiliations:** 1 Institut de Neuroscience des Systèmes, UMR Inserm 1106, Aix-Marseille Université, Faculté de Médecine, 27 Bd Jean Moulin, 13005 Marseille, France

## 

Epileptic seizures are characterized dynamically on multiple scales in space and time. Understanding the relations between components within and across those scales is among the fundamental goals of computational neuroscience [1,2], together with the analysis of the dynamical repertoire of such a neural system [3,4]. Previous research in this field has developed mathematical models that can reproduce seizure dynamics as either autonomous or driven processes [5]. In this study, we start from a phenomenological model displaying features of electrical epileptic neural activity, and we move towards a more biologically realistic network of neurons, while keeping track of the dynamical repertoire exhibited by the system [6]. Our system is composed of two neuronal populations, characterized by fast excitation and slow inhibition. The first one is modeled by 3-dimensional Hindmarsh-Rose oscillators, while the other is modeled by 2-dimensional Morris-Lecar oscillators. Both pools of fast and slow dynamics do not require manual drive in order to exhibit a repertoire of regimes that are commonly encountered in epileptic brain activity. We systematically run numerical parameter space exploration at each scale before going down to a lower scale with increased complexity.

Through this framework, we investigate plausible biological causation of epileptic dynamics such as electrical and chemical synaptic connectivity, excitation/inhibition ratios, and combine them to experimental data recorded from rodent hippocampus. This multiscale architecture allows to (i) perform automatic dynamical analysis of a particular neural system, (ii) characterize the level of biological accuracy required to portray epi-phenomena, and (iii) design new experiments to comprehend seizure mechanisms. We discuss the validity of our results with regard to both biologically and computationally oriented investigations that have been performed on the topic to provide a line of thoughts for future research.
